# Survey of the Quality of Experimental Design, Statistical Analysis and Reporting of Research Using Animals

**DOI:** 10.1371/journal.pone.0007824

**Published:** 2009-11-30

**Authors:** Carol Kilkenny, Nick Parsons, Ed Kadyszewski, Michael F. W. Festing, Innes C. Cuthill, Derek Fry, Jane Hutton, Douglas G. Altman

**Affiliations:** 1 The National Centre for the Replacement, Refinement and Reduction of Animals in Research, London, United Kingdom; 2 Warwick Medical School, University of Warwick, Coventry, United Kingdom; 3 Pfizer Global Research and Development, Groton, Connecticut, United States of America; 4 Animal Procedures Committee, London, United Kingdom; 5 School of Biological Sciences, University of Bristol, Bristol, United Kingdom; 6 Animals Scientific Procedures Inspectorate, Home Office, Shrewsbury, United Kingdom; 7 Department of Statistics, University of Warwick, Coventry, United Kingdom; 8 Centre for Statistics in Medicine, University of Oxford, Oxford, United Kingdom; University of Edinburgh, United Kingdom

## Abstract

For scientific, ethical and economic reasons, experiments involving animals should be appropriately designed, correctly analysed and transparently reported. This increases the scientific validity of the results, and maximises the knowledge gained from each experiment. A minimum amount of relevant information must be included in scientific publications to ensure that the methods and results of a study can be reviewed, analysed and repeated. Omitting essential information can raise scientific and ethical concerns. We report the findings of a systematic survey of reporting, experimental design and statistical analysis in published biomedical research using laboratory animals. Medline and EMBASE were searched for studies reporting research on live rats, mice and non-human primates carried out in UK and US publicly funded research establishments. Detailed information was collected from 271 publications, about the objective or hypothesis of the study, the number, sex, age and/or weight of animals used, and experimental and statistical methods. Only 59% of the studies stated the hypothesis or objective of the study and the number and characteristics of the animals used. Appropriate and efficient experimental design is a critical component of high-quality science. Most of the papers surveyed did not use randomisation (87%) or blinding (86%), to reduce bias in animal selection and outcome assessment. Only 70% of the publications that used statistical methods described their methods and presented the results with a measure of error or variability. This survey has identified a number of issues that need to be addressed in order to improve experimental design and reporting in publications describing research using animals. Scientific publication is a powerful and important source of information; the authors of scientific publications therefore have a responsibility to describe their methods and results comprehensively, accurately and transparently, and peer reviewers and journal editors share the responsibility to ensure that published studies fulfil these criteria.

## Introduction

Scientific progress is driven by developing and testing novel hypotheses. Investigating these new ideas using appropriately and robustly designed experiments is fundamental to this process. The entire scientific community is also equally reliant on published research being transparently and accurately reported. Critical appraisal of scientific publications, for instance by peer review, is only possible if the methods and results of the studies are comprehensively reported. Accurate and transparent reporting is therefore vital to allow the reader to assess the methods of the study, and the reliability and importance of the scientific findings. This is particularly necessary for scientific research using animals, as poorly designed experiments and reporting omissions can raise both ethical and scientific concerns.

The National Centre for the Replacement, Refinement and Reduction of Animals in Research (NC3Rs), established by the UK government in 2004, is an independent scientific organisation dedicated to finding innovative solutions to replace animals in research with non-animal alternatives, reduce the number of animals used in experiments, and minimise suffering and improve animal welfare by refining husbandry and procedures (the 3Rs). It is widely accepted that applying the 3Rs to experiments using animals is consonant with good scientific practice [1], [2]. Well designed experiments using sufficient animals to achieve a scientific objective, together with an appropriate statistical analysis, enable researchers to increase the robustness and validity of their experimental results, maximising the knowledge gained from each experiment whilst minimising the number of animals used.

In order to assess the scope for improved experimental design, statistical analysis and reporting, and to further the implementation of the 3Rs, the NC3Rs has carried out a systematic survey of the quality of reporting, experimental design and statistical analysis of recently published biomedical research using laboratory animals. This paper reports the main findings and conclusions of this survey.

## Results

### Included Studies

**Figure 1:**
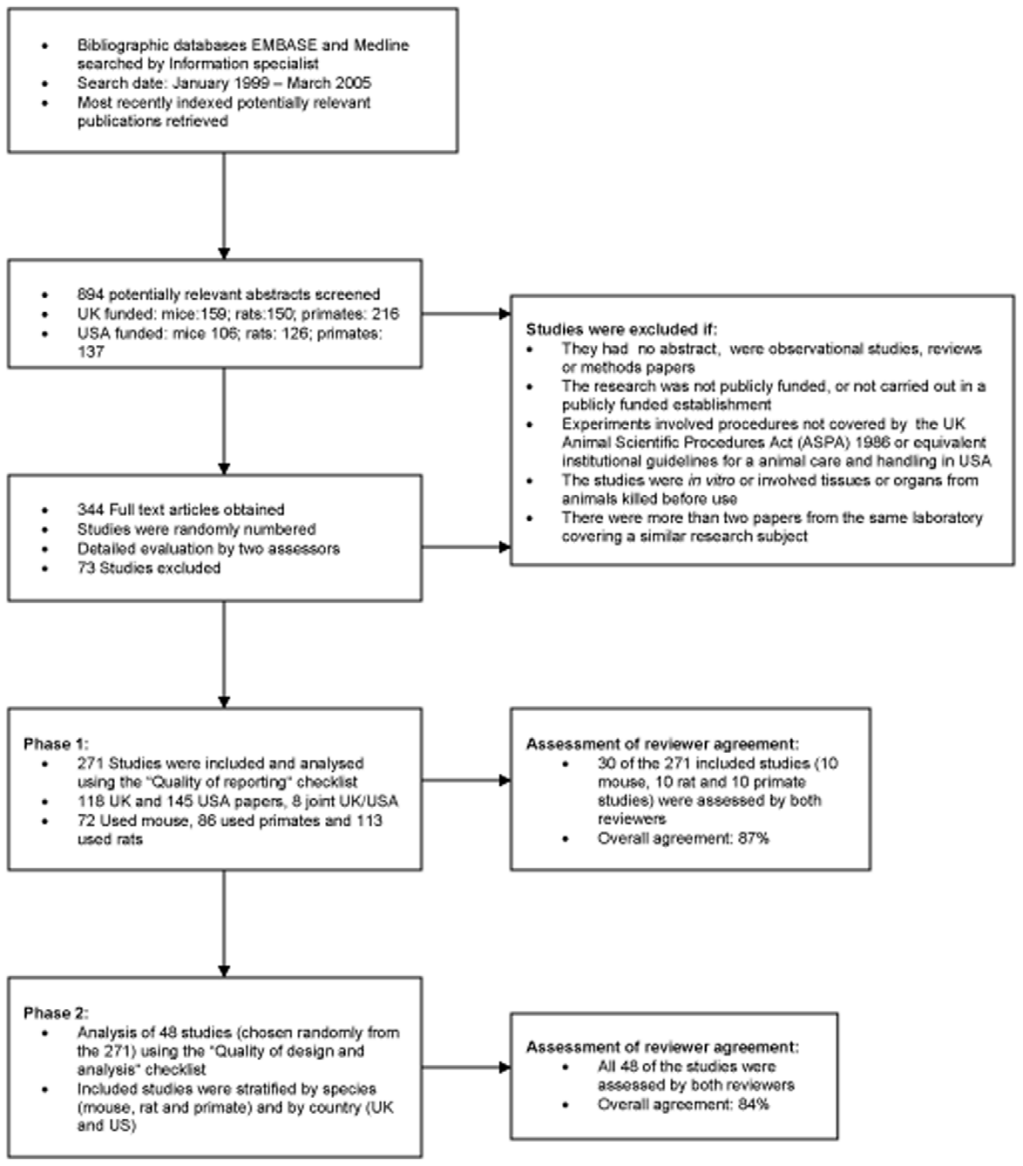
Flow diagram summarising the survey methods.

A systematic search of the Medline and EMBASE databases was carried out to identify potentially relevant scientific papers published between January 1999 and March 2005 reporting original research on live rats, mice and non-human primates (referred to hereafter as ‘primates’) (see Methods and Figure 1). Rodents are the most widely used animals and primates are the most ‘ethically sensitive’ group. From approximately 170,000 publications identified in the electronic search we selected 894 of the most recently indexed abstracts (see Methods). We chose the most recently indexed papers from all the papers identified in the search as an unbiased way of selecting the publications. We rejected 550 abstracts which did not meet our strict inclusion/exclusion criteria; 344 papers were chosen for full text analysis and closer scrutiny as to whether they met the inclusion criteria. An upper sample size limit of 300 full text papers (approximately 50 papers for each of the three taxa and two countries) was set prior to the database search, based on pragmatic considerations including the time taken to read each paper in sufficient detail to make a thorough, accurate and reliable assessment. The final sample consisted of 271 papers; 72 studies reporting experiments using mice, 86 using primates and 113 using rats; 118 reported research carried out in the UK, 145 in the USA and 8 carried out jointly in both countries (see Figure 1). Almost all (99%; 269/271) of the papers assessed were published between 2003 and 2005.

The search identified studies covering a wide variety of experimental fields, including behavioural and diet studies, drug and chemical testing, and immunological experiments (see Table 1), published in a comprehensive range of journals, covering a wide range of research areas, and funded by a number of funding bodies in the UK and USA including – but not limited to – the MRC, BBSRC, Wellcome Trust, and the NIH (see Table 2). This study was co-funded by two publicly funded bodies, the UK NC3Rs and the US National Institutes of Health/Office of Laboratory Animal Welfare (NIH/OLAW); we therefore decided to limit our included studies to publicly funded research carried out in the USA and the UK.

**Table 1:** Number of papers classified into general type of treatment procedure described in the study.

Species	Behaviour-Diet	Drug-Chemical	Immunization-Infection	Surgical	Other	Total
**Mouse (n = 72)**	6	14	29	2	21	72
**Primate (n = 86)**	30	14	13	15	14	86
**Rat (n = 113)**	17	46	6	25	19	113
**All (n = 271)**	53	74	48	42	54	271

**Table 2:** Number of studies reporting funding source classified by main funding body.

Funding Source	Number of Papers (n = 271)	Percentage
**US research**	116	43
**UK research**	76	28
**UK charity**	18	7
**US charity**	10	4
**Other**	23	8
**Unknown**	28	10†

† Of all the studies assessed, 10% (28/271) did not report their funding source(s).

The main experiment reported in each publication was identified and detailed information was collected on objective measures such as the numbers and characteristics of the animals used including the species, strain, age, sex, and weight. Details of the experimental design such as the size and number of experimental groups, how animals were assigned to experimental groups, how experimental outcomes were assessed, what statistical and analytical methods were used, were also recorded. This information was collected in two distinct stages. In phase 1, data were collected from all 271 papers, and in phase 2, a random sub-sample of 48 papers (stratified by animal and by country of origin; i.e. 8 papers×3 species×2 countries) was chosen from the 271 papers evaluated in phase 1, and assessed in more detail (see Methods). The majority of results reported here were based on the complete sample of 271 papers; where this was not the case the sample number is indicated in the text.

### Quality of Reporting

The survey's first question addressed the fundamental premise of each scientific publication. A clear statement of the objective of the study, or the main hypothesis being tested, was described in the introduction by 95% of the 271 publications; the remaining 5% of the studies either did not describe the purpose of the study at all, or it was not clear to the assessors (see Table 3). In 6% of all 271 studies surveyed it was unclear whether one, or more than one, experiment was being described (see Table 4). The experimental unit (e.g. a single animal or a group of animals) was not clearly identified in 13% of the 48 studies assessed in more detail (phase 2) (see Table 5). The species (in the case of primates) or strain of animal used was reported by 99% of all 271 studies assessed (see Table 6), with 74% of all studies reporting the sex of the animals (see Table 7). Only 43% of all 271 studies reported the age of the animals and 46% reported their weight; some papers reported both weight and age (13%), whilst 24% reported neither (see Table 8).

**Table 3:** Number of studies stating the purpose of the study in the introduction.

Species	No	Yes	Unclear	Yes (%)
**Mouse (n = 72)**	3	66	3	92
**Primate (n = 86)**	3	80	3	93
**Rat (n = 113)**	1	112	0	99
**All (n = 271)**	7	258	6	95†

† 95% (258/271) of all studies stated the purpose of the study in the introduction; 3% (7/271) did not and in 2% (6/271) the purpose of the study was unclear to the assessors.

**Table 4:** Number of experiments reported in each study.

Species	1	2	≥3	Unclear	1 (%)	≥2 (%)	Unclear (%)
**Mouse (n = 72)**	38	8	16	10	53	33	14
**Primate (n = 86)**	67	8	8	3	78	19	4
**Rat (n = 113)**	80	15	15	3	71	27	3
**All (n = 271)**	185	31	39	16	68	26	6†

† 68% (185/271) of all studies reported the results of one experiment, but in 6% (16/271) of all the studies it was unclear whether one or more experiments were being described.

**Table 5:** Number of studies that clearly identified the experimental unit.

Species	Unclear†	No†	Yes†	No or Unclear (%)
**Mouse (n = 16)**	1	1	30	6
**Primate (n = 16)**	3	2	27	16
**Rat (n = 16)**	0	5	27	16
**All (n = 48)**	4	8	84	13*

† In phase 2 of the survey all 48 studies were assessed independently by two assessors, therefore numbers in each row sum to twice the number of studies.

* The experimental unit (e.g. a single animal or a group of animals) was not clearly identified in 13% (12/96) of the studies assessed.

**Table 6:** Number of studies reporting the species or strain of the animals.

Species	No	Yes	Yes (%)
**Mouse (n = 72)**	0	72	100
**Primate (n = 86)**	0	86	100
**Rat (n = 113)**	2	111	98
**All (n = 271)**	2	269	99†

† The species (in the case of primates) or strain of animal used was reported by 99% (269/271) of all the studies surveyed.

**Table 7:** Number of studies reporting the sex of the animals.

Species	No	Yes	Unclear	Yes (%)
**Mouse (n = 72)**	24	47	1	65
**Primate (n = 86)**	30	55	1	64
**Rat (n = 113)**	15	98	0	87
**All (n = 271)**	69	200	2	74†

† 74% (200/271) of all studies reported the sex of the animals used in the main experiment.

**Table 8:** Number of studies reporting the age and weight of the animals.

Species	Age	Weight			
		Unclear	No	Yes	No. of papers
**Mouse (n = 72)**	**No**	0	23	5	28
	**Yes**	0	41	3	44
**Primate (n = 86)**	**No**	0	33	17	50
	**Yes**	1	19	16	36
**Rat (n = 113)**	**No**	0	10	66	76
	**Yes**	1	19	17	37
**All (n = 271)**	**No**	0	66	88	154
	**Yes**	2	79	36	117

† 43% (117/271) of the studies reported the age of the animals used in the main experiment; 46% (124/271) of studies reported the weight of the animals used in the main experiment; 24% (66/271) of the papers reported neither the weight nor the age of the animals used, whilst 13% (36/271) reported both weight and age.

In 4% of the 271 included publications, the number of animals used in the main experiment assessed was not reported anywhere in the methods or the results sections (see Table 9). None of the 48 studies assessed in more detail that did report animal numbers, discussed how the sample size was chosen (see Table 10). In 35% (69/198) of the papers that reported animal numbers in the methods section, the number of animals was either not reported in the results section, was unclear, or was different from that reported in the methods. In the majority of cases the number of animals reported in the results section was larger than in the methods section although in some papers the reverse was true (see Table 11).

**Table 9:** Number of studies reporting animal numbers in the methods and results sections.

Species	Methods section	Results section		
		No record	Estimated number	Exact number
**Mouse (n = 72)**	**No record**	6	15	19
	**Estimate**	2	2	2
	**Exact**	9	1	16
**Primate (n = 86)**	**No record**	0	0	6
	**Estimate**	1	0	0
	**Exact**	18	1	60
**Rat (n = 113)**	**No record**	6	12	10
	**Estimate**	5	1	4
	**Exact**	21	5	49
**All (n = 271)**	**No record**	12†	27	35
	**Estimate**	8	3	6
	**Exact**	48	7	125

† 4% (12/271) of all included studies had no record of animal numbers in either the methods or results sections.

**Note:** Studies were assessed according to whether the exact number of animals used was reported (e.g. 50 rats divided into 5 treatment groups comprising 10 rats each), the number of animals could be estimated (e.g. 50 rats divided into 5 groups or treatments comprised 8–12 rats) or the number of animals was not clearly stated (e.g. treatments were applied to 5 groups of rats).

**Table 10:** Number of studies that explained the sample size.

Species	Unclear†	No†	Yes†	Yes (%)
**Mouse (n = 16)**	0	32	0	0
**Primate (n = 16)**	1	31	0	0
**Rat (n = 16)**	0	32	0	0
**All (n = 48)**	1	95	0	0

† In phase 2 of the survey all 48 studies were assessed independently by two assessors, therefore numbers in each row sum to twice the number of studies assessed for each species.

**Table 11:** Number of animals reported in methods and results sections for each study.

Species	Methods section	Results section					
		0–9	10–19	20–29	30–39	40+	??†
**Mouse (n = 72)**	**0–9**	2	1	1	0	0	0
	**10–19**	0	3	0	1	0	2
	**20–29**	0	0	4	0	0	3
	**30–39**	0	0	0	2	0	2
	**40+**	0	0	0	0	7	5
	**??**	4	8	2	5	15	5
**Primate (n = 86)**	**0–9**	37	0	0	0	0	9
	**10–19**	0	12	0	0	0	6
	**20–29**	0	0	4	0	0	4
	**30–39**	0	0	0	4	0	0
	**40+**	0	0	0	0	4	0
	**??**	6	0	0	0	0	0
**Rat (n = 113)**	**0–9**	3	0	0	0	1	4
	**10–19**	0	13	0	1	0	6
	**20–29**	0	1	14	0	0	3
	**30–39**	0	0	1	5	0	5
	**40+**	0	2	0	3	15	8
	**??**	2	5	5	3	7	6
**All (n = 271)**	**0–9**	40	1	1	0	0	13
	**40+**	0	2	0	3	26	13
	**??**	12	13	7	8	22	11

† The ‘??’ symbol indicates that the number of animals was not clear or not reported. In 35% (69/198) of the papers which did report animal numbers, the numbers differed between the methods and the results sections of the paper.

In order to investigate the proportion of papers that had multiple reporting omissions and to provide an overall assessment of the quality of reporting, we identified those papers which clearly stated the study hypothesis, reported three animal characteristics (sex, strain and weight or age), and also reported the number of animals used; 59% of all 271 papers reported all this information (see Table 12).

**Table 12:** Number of studies reporting the study hypothesis, three animal characteristics and the number of animals used.

Species	Characteristics†	Hypothesis clearly stated	Animal numbers Not reported	Reported
**Mouse**	**No**	**No**	0	3
		**Yes**	3	28
	**Yes**	**No**	1	2
		**Yes**	2	33
**Primate**	**No**	**No**	0	5
		**Yes**	0	40
	**Yes**	**No**	0	1
		**Yes**	0	40
**Rat**	**No**	**No**	0	0
		**Yes**	2	20
	**Yes**	**No**	0	1
		**Yes**	4	86
**All**	**No**	**No**	0	8
		**Yes**	5	88
	**Yes**	**No**	1	4
		**Yes**	6	159*

† Sex, strain and either weight or age.

* 59% (159/271) of all papers clearly stated the study hypothesis, reported three animal characteristics (sex, strain and weight or age), and also reported the number of animals used.

### Quality of Experimental Design

Next we assessed the quality of experimental design, and in particular, how many papers had incorporated measures to reduce bias such as randomisation and blinding. Formal randomisation is a process used to allocate animals to experimental groups and is carried out to avoid any bias in assigning the animals to the treatment groups, making it more likely that the groups are comparable [2], [3]. The aim is to ensure that, as far as possible, any differences in outcome measures observed between the groups can be ascribed purely to the experimental procedures. Random selection is not the same as haphazard selection; a systematic, physical approach such as tossing a coin or using a table of random numbers or a computer to pick numbers randomly, is necessary for this process. Random allocation of animals to experimental groups was reported in 12% of all 271 studies in the sample (see Table 13). Of the studies which reported using randomisation, 9% (3/33) provided details of the method used.

**Table 13:** Number of studies that reported using randomisation.

Species	No	Yes	Yes (%)
**Mouse (n = 72)**	67	5	7
**Primate (n = 86)**	83	3	7
**Rat (n = 113)**	89	24	20
**All (n = 271)**	239	32	12†

† Random allocation of animals to experimental groups was reported in only 12% (32/271) of all the studies in the survey.

Qualitative scoring of an experimental observation or result by a researcher often involves a subjective assessment or judgement, and as such is more susceptible to bias than quantitative (numeric) measures (e.g. weight). Blinding, where the researcher does not know which treatment the animal has received when judging an experimental result, is an effective way of minimising this bias [3]. Only 14% (5/35) of all papers in the whole sample that used qualitative scores also reported that they used blinding (see Table 14).

**Table 14:** Number of studies that used qualitative scores reporting blinding.

Species	Blinding	No. of qualitative scores		
		0	1	≥2
**Mouse (n = 72)**	No	60	5	4
	Yes	3	0	0
**Primate (n = 86)**	No	74	1	6
	Yes	1	1	3
**Rat (n = 113)**	No	91	4	10
	Yes	7	1	0
**All (n = 271)**	No	225	10	20
	Yes	11	2	3

Blinding would usually be expected to be used, and reported, in those studies where qualitative scores were used. The percentage of papers which reported using blinding where one or more qualitative variables were used; All = 14% (5/35); Mouse = 0% (0/9); Primate = 36% (4/11) and Rat = 7% (1/15).

A design factor is an experimental variable (e.g. treatment) to which an animal is allocated by a researcher, ideally at random. Other variables that may influence the effect(s) of treatment(s) but that cannot be randomly assigned to the animals (such as sex or strain) can also be chosen by the researcher. Factorial and stratified experimental designs allow combinations of two or more design factors to be evaluated in one experiment at the same time in the same animals. These types of experimental design are efficient ways of maximising the information gained from each experiment, and can reduce the overall number of animals used whilst increasing the strength of the scientific findings [4], [5]. Two or more design factors are necessary for a factorial design to be used. We found that only 62% (75/121) of all the experiments assessed that were amenable to a factorial design (and analysis) reported using one (see Table 15). Hence it seems that a large number of the studies assessed did not make the most efficient use of the available resources (including the animals), by using the most appropriate experimental design.

**Table 15:** Number of studies using a factorial design.

Species	Unclear	No	Yes	Yes (%)
**Mouse (n = 36)** †	0	11	25	69
**Primate (n = 26)** †	0	12	14	54
**Rat (n = 59)** †	0	23	36	61
**All (n = 121)** †	0	46	75	62

† Number of studies that used two or more design factors.

Overall 62% (75/121) of all studies that had two or more design factors reported using a factorial design.

### Quality of Statistical Analysis

Statistical methods were used to analyse the data in 91% of all 271 studies; ANOVA and t-tests were the methods used most frequently. However in 4% (10/247) of the studies that used statistics, it was unclear what statistical method had been used, i.e. a p-value or statistical significance was indicated, but no other methodological details were reported (see Table 16). Further analysis showed that overall only 70% (174/247) of papers that used a statistical method described the method employed, and also presented the numerical results with a measure of variation (e.g. standard deviation) or an error measure (e.g. standard error of the mean [SEM] or confidence interval [CI]; see Table 17). Of the 48 studies assessed in more detail, 39 used and described a statistical method, 34 of these (87%) were judged by the two statistical assessors to have used an appropriate statistical method, however in the remaining 5 papers there was insufficient information reported in the publication to be able to make this judgement (see Table 18).

**Table 16:** Number of studies where the statistical method was not reported or was unclear.

Species	Methods reported	Unclear or not reported	Unclear or not reported (%)
**Mouse (n = 60)** †	56	4	7
**Primate (n = 76)** †	74	2	3
**Rat (n = 111)** †	107	4	4
**All (n = 247)** †	237	10	4*

† Number of studies where the statistical methods were not reported or not clear.

* In 4% (10/247) of the studies which used statistics, it was unclear or uncertain what statistical method had been used, i.e. a p-value or statistical significance was indicated, but no other methodological details were reported.

**Table 17:** Number of papers which used statistical methods reported the method used and also used an error measure.

Species	Statistical methods described†	Statistical method(s) used	Error measures‡	
			Not used	Used
**Mouse**	**No**	**No**	4	7
		**Yes**	3	10
	**Yes**	**No**	1	0
		**Yes**	6	41
**Primate**	**No**	**No**	8	1
		**Yes**	8	15
	**Yes**	**No**	1	0
		**Yes**	9	44
**Rat**	**No**	**No**	0	2
		**Yes**	7	12
	**Yes**	**No**	0	0
		**Yes**	3	89
**All**	**No**	**No**	12	10
		**Yes**	18	37
	**Yes**	**No**	2	0
		**Yes**	18	174*

† Statistical methods described in materials and methods section of paper.

‡ Standard error of the mean, confidence interval, standard deviation or other error measurement.

* 70% (174/247); 91% (247/271) of all included studies used statistical methods to analyse the data; 17% (46/267) of the studies, that presented numerical data, did not present a measure of variation (e.g. standard deviation) or uncertainty (e.g. standard error of the mean [SEM] or confidence interval [CI]).

**Table 18:** Number of studies that use an appropriate statistical method.

Species	Unclear	No	Yes	Yes (%)
**Mouse (n = 10)** †	4	0	16	80
**Primate (n = 13)** †	4	0	22	85
**Rat (n = 16)** †	1	1	30	94
**All (n = 39)** †	9	1	68	87*

† Numbers of studies in phase 2 that were assessed independently by two assessors and used a statistical method; numbers in each row sum to twice the number of studies.

* Of the 48 studies assessed in more detail, and that used and described a statistical method, 87% (34/39) were judged to have used a correct statistical method, and in 12% (5/39) of the papers assessed there was insufficient information reported in the publication to be able to make this judgement.

Although almost all (99%) of the 271 included studies presented numerical data (see Table 19), only about half of the 48 studies assessed in more depth, stated the number of experimental units (e.g. individual animals or cages of animals) in all figures and tables [6] (see Table 20). These omissions make it difficult for the reader to assess and interpret the results. Only 8% of the 48 studies assessed presented raw data for individual animals (see Table 21). Reporting raw data, particularly in studies where only a small number of animals are used, is valuable as it allows a more complete and independent assessment of the results.

**Table 19:** Number of studies presenting numerical data.

Species	No	Yes	Unclear	Yes (%)
**Mouse (n = 72)**	1	71	0	99
**Primate (n = 86)**	1	84	1	98
**Rat (n = 113)**	0	112	1	99
**All (n = 271)**	2	267	2	99†

† 99% (267/271) of the included studies presented numerical data. The study was scored “Yes” if numerical data were presented graphically, in tabular form or in the text, either for each animal or by treatment group.

**Table 20:** Number of studies clearly stating the numbers of experimental units (e.g. individual animals or cages of animals) in all figures and tables.

Species	Unclear†	No†	Yes†	Yes (%)
**Mouse (n = 16)**	1	7	24	75
**Primate (n = 16)**	1	20	11	34
**Rat (n = 16)**	0	20	12	38
**All (n = 48)**	2	47	47	49

† In phase 2 of the survey all 48 studies were assessed independently by two assessors, therefore numbers in each row sum to twice the number of studies.

* 49% (47/96) of the 48 studies assessed clearly stated the number of experimental units in all figures and tables.

**Table 21:** Number of studies reporting raw data for individual animals.

Species	Unclear†	No†	Yes†	Yes (%)
**Mouse (n = 16)**	1	29	2	6
**Primate (n = 16)**	1	27	4	13
**Rat (n = 16)**	0	30	2	6
**All (n = 48)**	2	86	8	8*

† In phase 2 of the survey all 48 studies were assessed independently by two assessors, therefore numbers in each row sum to twice the number of studies.

* Only 8% (4/48) of the 48 studies presented raw data for individual animals.

## Discussion

The NC3Rs survey has provided a detailed analysis of both the quality of the reporting and the quality of the experimental design and statistical analysis of experimental research using laboratory animals. The survey has identified a number of issues – particularly reporting omissions.

Every study faces a trade-off between maximising power within the study itself (internal validity) by minimising sample heterogeneity and maximising the generalisability of the findings (external validity). The number of papers assessed in this survey is approximately twice the number of studies included in similar surveys published to date (133 and 149 papers respectively) [7], [8], and as our results have indicated, was of sufficient size to be able to identify important problems with reporting, experimental design and statistical analysis.

Our study was carefully designed to ensure that the sample was representative of the target literature. The search strategy, which included species names, will have selected for a subset of papers, i.e. those that at least reported the species of animal used. Whilst our findings apply to this sample of papers, our results may in fact underestimate the extent of the reporting omissions. It is highly unlikely that our search terms or inclusion/exclusion criteria would have biased the sample to include a disproportionate number of poor quality publications from lower ranking journals. In fact, the search retrieved papers from a range of publication years (1999 – 2005), covering a wide variety of research areas, and an extensive range of journals across the impact factor spectrum, including *Nature* and *Science*. Whilst it would be useful to know if there is a relationship between the quality of the papers surveyed and the impact factors of the journals they were published in, this analysis was not in the remit of this survey.

### Statement of Hypothesis

Scientific papers should report sufficient relevant information about the experimental objectives, animal characteristics, experimental methods used and results obtained, in order to critically assess the findings and both the scientific and ethical implications of a study, or to allow the work to be repeated. Surprisingly, some of the studies surveyed either did not describe the purpose of the study at all or it was unclear to the assessors, and thus presumably also to any non-specialist reader. In addition, in some of the studies surveyed it was unclear whether one or more experiments were being described, and the experimental unit (e.g. a single animal or a group of animals) was not clearly identified.

### Animal Characteristics

Many of the studies surveyed omitted details about the strain, sex, age and weight of the animals used. These are all factors that can potentially influence experimental results and are therefore scientifically important [9]–[11]. This information is generally readily available to researchers and can be succinctly described, so it is unclear why omitting these essential details is so prevalent.

Many journals offer supplementary online space (generally unlimited) not only for methodological information but also for additional results and tables. This information resource was considered, where it was available, for the papers surveyed. The availability of this resource negates the argument that the lack of detail in published papers is primarily due to a lack of space. Studies have found that some experimental details (such as chemical interactions and equipment) are extensively discussed in the body of the paper, whilst information about animal characteristics, sample sizes etc are scantily provided or are absent [10], [11].

### Animal Numbers

In some of the included publications, the number of animals used was not reported anywhere in the methods or the results sections. Reporting animal numbers is essential so that the biological and statistical significance of the experimental results can be assessed or the data re-analysed, and is also necessary if the experimental methods are to be repeated. Crucially, none of the studies assessed in more detail discussed how the sample size was chosen.

Power analysis or other very simple calculations, which are widely used in human clinical trials and are often expected by regulatory authorities in some animal studies, can help to determine an appropriate number of animals to use in an experiment in order to detect a biologically important effect if there is one [3], [12]. This is a scientifically robust and efficient way of determining animal numbers and may ultimately help to prevent animals being used unnecessarily. Many of the studies that did report the number of animals used reported the numbers inconsistently between the methods and results sections. The reason for this is unclear, but this does pose a significant problem when analysing, interpreting and repeating the results.

### Experimental Design

The assessment of experimental design found that random allocation of animals to treatment groups was reported in only a very small proportion of all the studies surveyed. Randomisation reduces selection bias, increases the validity of the findings and, in principle, is always an appropriate and desirable aspect of good experimental design when two or more treatments are compared [3]. Randomisation should also extend to cage placement within rooms in the animal house and the order in which experimental treatments and assessments of the animals/cages are made. Randomised block designs – where the experimental animals are first divided into groups before the groups are randomly assigned to a treatment group – can be used to introduce variation in the groups of animals (e.g. sex, age, severity of disease) in a controlled way without the need for larger numbers of animals [13].

We cannot rule out that some of the studies surveyed may have used randomisation where appropriate, but did not report using it. If this was the case, then this kind of reporting omission can easily be rectified. But if not, incomplete reporting masks potentially flawed experimental methods.

“*When humans have to make observations there is always the possibility of bias*” [14]. Blinded assessment, where appropriate, minimises any bias in the qualitative scoring of subjective experimental observations, improving the rigour of the experimental method and the scientific validity of the results obtained, and yet blinding is rarely reported as being performed [3]. It cannot be ruled out that a proportion of the studies may indeed have used blinding but did not report it.

Reviews of animal research in the field of emergency medicine found that studies which did not use randomisation and blinding to reduce bias when comparing two or more experimental groups, were significantly more likely to find a difference between the treatment groups [15], [16]. Those studies that did incorporate these measures gave a lower estimate of treatment efficacy, meaning that the treatment effects were more likely to be accurately estimated. These findings indicate that experimental designs which minimise bias have implications for the robustness of scientific results and, in biomedical research, the suitability of these animal studies for translation into clinical trials.

### Statistical Analysis

Statistical methods are important for calculating the degree of confidence in, for example, the reproducibility and general validity of experimental results, and were used and reported by the majority of studies. The majority of the studies that used and described a statistical method were judged to have used a correct statistical method. Whilst the majority of papers that used a statistical method described it and reported the numerical results with an error measure, many papers did not. Reporting the statistical method used together with an indication of the measure of variation or uncertainty is essential for interpreting any results, and has implications for the reliability and generalisability of the findings to other species and systems (external validity) [3], [17].

Our findings indicate that there are problems both with the transparency of reporting and the robustness of the statistical analysis of almost 60% of the publications surveyed. In many papers, due to the lack of information detailing the statistical methods it was difficult to judge whether or not the statistical analysis were appropriate, or if data had been efficiently extracted and analysed.

### Previous Surveys

These issues are not new, as previous surveys of publications describing animal research and assessing specific aspects of experimental design, statistical analysis and reporting, have shown [7], [8], [11], [18], [19]. One survey of animal research published in the Australian Veterinary Journal, found that 30% of the papers surveyed had experimental design flaws including a lack of randomisation, whilst 45% had used suboptimal methods of statistical analysis and contained calculation errors [7]. Data omissions and errors in presentation were other common findings. The author concluded that the quality of reporting, experimental design and statistical analysis in reports of scientific research could be improved.

The problems with experimental design and reporting that we have identified are also in line with similar reviews of the literature in various other scientific and clinical research areas [18]–[25]. In these research areas too, the quality of reporting and experimental design has been found wanting. The entire scientific community is reliant on published experiments being appropriately designed and carried out, and accurately and transparently reported, as this has implications for the scientific validity of the results.

### Reporting Guidelines

Standards developed for reporting clinical research have improved the quality and transparency of reporting of clinical trials and have been adopted by many leading medical journals as part of their instructions to authors [26], [27]. Reporting guidelines have also been developed for other specific research areas [28]–[33]. However, most biomedical journals currently provide little or no guidance about how to report research using animals apart from the ethical considerations regarding the procedures used. We believe that there is a need to develop reporting standards specifically for research using animals, and to provide guidance on the relevant information that should be included, with the aim of enhancing the transparency of reporting and encouraging both researchers, and those journals responsible for publishing this research, to adopt and adhere to them.

### Conclusion

This is the largest and most comprehensive survey of this kind carried out to date. We provide evidence that many peer-reviewed, animal research publications fail to report important information regarding experimental and statistical methods. Whilst our findings are limited to experimental studies using rodents and primates carried out in UK and US laboratories, this is the statistical population that dominates the biomedical research literature, so our results are important and indeed, indicate cause for concern.

Scientific publication is the method by which research has traditionally been described and the results communicated and it remains a powerful and important source of information. The authors of scientific publications therefore have a responsibility to describe their experimental and statistical methods and results comprehensively, accurately and transparently, and journal editors share the responsibility to ensure that published studies fulfil these criteria. This is particularly pertinent for research involving animals, as poorly designed and reported experiments raise ethical as well as scientific concerns. Whilst we recognise that in some studies, not all of the details we assessed (e.g. the sex of animals) will necessarily have an important impact on the overall findings, there are principles at stake – namely the transparency, reproducibility, and reliability of scientific publications. We are simply arguing for the inclusion of all relevant information that will allow a suitably skilled reader to assess, analyse, and repeat the study's findings.

There are many opportunities for the scientific community to improve both the experimental design and the quality of reporting of biomedical research using animals. Serious efforts are needed to improve both the quality of experimental design and the quality of reporting in order to make research articles better suited to the needs of readership. The NC3Rs has identified a number of ways of helping to make these improvements. Raising awareness that these problems exist will be the first step in tackling these fundamental issues. In addition, working with researchers, journal editors and funding bodies, the NC3Rs is building on the results of this survey by developing a set of reporting guidelines to assist researchers, journal editors and research funding bodies to take appropriate steps to improve the quality and transparency of reporting in the scientific publications with which they are associated.

## Methods

### Database Search for Published Studies

An information specialist searched the Medline and EMBASE databases for all potentially relevant English language scientific papers published between 1 January 1999 and 31 March 2005, reporting original research on live rats, mice and non-human primates (referred to hereafter as ‘primates’) carried out in publicly funded research establishments in the UK and the USA. (See supplementary online information for search terms).

### Search Strategy

Databases were searched using the following search terms:

1. exp MICE 14. Hominidae

2. usa.in. 15. 12 or 13

3. 1 and 2 16. 15 not 14

4. exp great britain17. Pan troglodytes

5. england.in.18. 16 or 17

6. uk.in.19. exp CEBIDAE

7. 4 or 5 or 620. exp MACACA

8. 1 and 721. exp Papio

9. exp RATS22. 18 or 19 or 20 or 21

10. 9 and 223. 22 and 7

11. 9 and 724. 22 and 2

12. PRIMATES25. 3 or 8 or 10 or 11 or 23 or 24

13. Haplorhini

### Sample Size

An upper limit on the number of papers that would be included in the survey was set at 300 – made up of approximately 50 papers for each of three species and two countries. This limit was based on pragmatic considerations that included the time taken to assess and extract information from each publication. The sample size for surveys such as this is not normally based on formal statistical considerations, as there are no primary hypotheses being tested. There was therefore no need to formally power this study.

### Selecting Published Studies

A sample of the most recently indexed abstracts was selected from the total number of potentially relevant publications identified in the database search. We chose the most recently indexed papers from all the papers identified in the search as an unbiased way of selecting the publications. When a journal is added to a database and becomes indexed, all previous issues are also indexed, enabling us to have a spread of publication years in the sample. The abstracts were appraised and publications were selected or rejected based on the exclusion criteria listed below (see Figure 1). The full texts of the remaining publications were obtained. Each potentially relevant full text was numbered within its country-species stratum and the exact reference of each paper recorded. Three digit random numbers were generated using MINITAB, and the six lists were re-ordered using the random numbers. This stratified randomisation procedure was carried out to minimise bias, to ensure the total sample was representative of the six subgroups (i.e. three species and two countries), and to allow analysis of each subgroup in addition to the overall sample. The first fifty papers for each species and country were considered from each of the six randomised lists. If a paper was not eligible, the next paper on the randomised list was taken. A second reviewer independently assessed the full texts of all the selected papers and finalised the list of included studies. Some further studies were excluded in this step.

### Inclusion Criteria

All relevant English language studies published between January 1999 and March 2005, reporting original scientific research carried out in UK or USA publicly funded research establishments and whose source(s) of funding were UK- or USA- publicly funded bodies such as universities, charities or other non-industry funding, such as the NIH, USPHS, MRC, BBSRC, etc., were included. Studies that had any commercial/industry funding were included only if the majority of the other funding sources were UK or USA public funding sources and the work was carried out in a UK or USA publicly funded research establishment. Studies that had any non-UK or non-USA public funding were included only if the majority of the other funding sources were from UK or USA public funding sources and the work was carried out in a UK or USA publicly funded research establishment. Studies whose funding source was not stated were included only if the research was carried out at a UK or USA publicly funded institution. Note was made that the funding source information was not reported.

We chose to limit our investigation to publicly funded research in the USA and UK because the funding for this study came from both US and UK publicly funded bodies, the two countries are highly influential in setting the scientific agenda, and because there should theoretically be no constraints on reporting publicly funded research for reasons of confidentiality or commercial sensitivity.

The survey was restricted to original scientific research studies using mice, rats, and primates. The experiments had to use live animals (including terminal anaesthesia) and state that they had used UK Animals [Scientific Procedures] Act 1986 (ASPA) licensed interventions, or equivalent USA institutional guidelines for animal care and use. Rodents are the most widely used animals and primates are the most high profile and ‘ethically sensitive’ group (for convenience primates are designated a species here). Other species or groups such as fish, birds, rabbits and guinea-pigs are either used in small numbers or in more specialised areas of research. The sample sizes for these species would have been too small to draw any strong inferences about the reporting standards in these research areas. In addition, every such study that was included would reduce the statistical power of the study for drawing inferences about reporting and experimental design standards studies involving more widely used species.

### Exclusion Criteria

Publications were excluded if industry/commercial funding was the sole source of funding, or if the research was solely funded by an organisation not based in the USA or UK. *In vitro* studies, studies using tissue from animals killed before use, or that did not involve experimental procedures/testing, technical or methodological papers not involving actual experiments using animals, review articles, genetics papers reporting linkages of genes, studies with no abstract, and brief communications with no methods, were also excluded. No more than two papers were included from any single laboratory to ensure that the survey results were not unduly influenced by the bad – or good – practice of one particularly productive laboratory.

### Unit of Analysis

The unit of analysis was the ‘main experiment’ reported in the paper. Many papers report the results of more than one experiment; accordingly, the number of experiments per paper was noted. For those studies that reported more than one experiment, the experiment that used the most animals was considered the ‘main experiment’. Details and results from the main experiment were used to complete the data collection sheets. Although the specific details described in this report relate to a single experiment assessed in each publication, the whole paper was searched for information relevant to that experiment, and to the way the experimental work was conducted and analysed in general.

### The Survey Process

The survey was carried out in two steps identified as phases 1 and 2.

#### Phase 1: quality of reporting

In phase 1, the full texts of the 271 included studies were divided equally between two assessors who were experienced statisticians (one from the UK and one from the USA). Assessor 1 analysed the even numbered papers, assessor 2 analysed the odd numbered papers extracting the relevant information to complete the Quality of Reporting checklist (see Supporting Information S1). Any supplementary online data associated with any of the included publications was accessed and analysed.

#### Phase 2: quality of experimental design and statistical analysis

In phase 2, a random sub-sample of 48 papers chosen from the 271 papers evaluated in phase 1, stratified by animal and by country (i.e. 8 papers×3 species×2 countries), was assessed. This number was selected as an appropriately sized sub-sample of the papers assessed in phase 1 based, as was the case for phase 1, on the time necessary to complete the very detailed reports. The statistical methods and analysis of the papers were assessed to determine whether the experimental design and the statistical analysis were appropriate. This involved the expert judgement of two statisticians, both of whom assessed all 48 papers using the Quality of Experimental Design and Analysis checklist (see Supporting Information S1). The main experiment was the same as that analysed in phase 1. Errors of omission were noted.

### Assessor Agreement

Any disagreements or differences in interpretation of the checklists were resolved by consultation and discussion with a third assessor and, where necessary, the relevant studies were re-analysed. To allow for possible discrepancies between the two assessments, in phase 2 the mean of the results from the two statisticians are reported in all data summary tables. Overall agreement between the assessors was assessed once during each phase of the survey – In phase 1 both assessors applied the relevant checklist to the same sub-set of 30 (of 271) papers and their analyses compared, and in phase 2, all 48 papers were used to assess agreement (see Figure 1).

## Supporting Information

Supporting Information S1 Survey Checklists
(0.17 MB PDF)
