# Supplementary material for: Survey of the Quality of Experimental Design, Statistical Analysis and Reporting of Research Using Animals
Source: PLoS One. 2009 Nov 30;4(11):e7824. doi: 10.1371/journal.pone.0007824 (PMC2779358; doi:10.1371/journal.pone.0007824)
Supplement: Supporting Information S1 — Survey Checklists (0.17 MB PDF) [file pone.0007824.s001.pdf]

Supporting Information – Checklists phase 1 and 2.

NC3Rs Survey of Experimental design and reporting

Quality of Reporting Checklist (Phase 1)

**Paper Number:**		**Assessor:**			**Date:**		
(1)	Is the purpose of the study stated in the introduction?				No	Yes	??
(2)	Number of experiments	1	2	3	4	>4	??
(3)	Sex						
	(a) Is sex reported?				No	Yes	??
	(b) Sex			M	F	Both	??
(4)	Strains (rodents) and species (primates)						
	(a) Is strain or species reported?				No	Yes	??
	(b) Number of strains or species reported?				1	>1	??
(5)	Age and Weight						
	(a) Is age recorded?				No	Yes	??
	(b) Is weight recorded (rodents)?				No	Yes	??
(6)	Experimental design						
	(a) Number of design factors						??
	(b) Number of other (non-design) factors						??
	(c) Number of treatment (design) groups						??
	(d) Is a factorial design used?				No	Yes	??
(7)	Type of treatment or procedure (state)						??
(8)	Type of experiment						
	(a) Between subject				No	Yes	??
	(b) Within subject				No	Yes	??
(9)	Animal number records (methods section)				Exact	Estimate	??
	(a) Total number of animals (methods section)						??
(10)	Animal number records (results section)				Exact	Estimate	??
	(a) Total number of animals (results section)						??
(11)	Is randomisation stated as used?				No	Yes	??
(12)	Are measurements taken blind?				No	Yes	??
(13)	Numerical data presented?				No	Yes	??
(14)	No. of dependent variables (outcomes)						
	(a) Qualitative	0	1	2	3	>3	??
	(b) Quantitative	0	1	2	3	>3	??
(15)	Statistical methods						
	(a) Reported in materials and methods section				No	Yes	??
	(b) Reported in results section				No	Yes	??
(16)	Method of statistical analysis						
	(a) None				No	Yes	??
	(b) Chi-squared / Fisher's exact test				No	Yes	??
	(c) T-test				No	Yes	??
	(d) Mann-Whitney / Wilcoxon				No	Yes	??
	(e) ANOVA				No	Yes	??
	(f) Correlation				No	Yes	??
	(g) Regression				No	Yes	??
	(h) Other/s (state)				No	Yes	??
	(i) Unknown / Uncertain				No	Yes	??
(17)	Primary outcomes presented with		SD	SEM	CI	None	??
(18)	Source of funding (state)						??

NC3Rs Survey of Experimental Design and Reporting		
**Quality of Analysis and Design Checklist (Phase 2)**		
**Paper Number:**	**Assessor:**	**Date:**

(1)	Are there clearly stated hypotheses?	No	Yes	??
(2)	Is the main experiment clearly distinguished?	No	Yes	??
(3)	Design methods			
	(a) Is the randomisation method stated?	No	Yes	??
	(b) Is blocking used?	No	Yes	??
(4)	Is the experimental unit correctly identified?	No	Yes	??
(5)	Is there evidence of pseudoreplication?	No	Yes	??
(6)	Sample size			
	(a) Is the sample size justified in the methods section?	No	Yes	??
	(b) Are there equal numbers per treatment group?	No	Yes	??
	(c) If not is this justified?	No	Yes	??
(7)	Sizes of treatment groups			
	(a) Number of animals per treatment group (methods section)			??
	(b) Number of animals per treatment group (results section)			??
(8)	Data completeness			
	(a) Are data omitted?	No	Yes	??
	(b) Are data not collected?	No	Yes	??
	(c) Are omissions and data not collected explained?	No	Yes	??
(9)	Are numbers of units stated in all figures and tables?	No	Yes	??
(10)	Subjective scores			
	(a) Are subjective scores used (histology)?	No	Yes	??
	(b) If so are they done blind?	No	Yes	??
(11)	Are inferential methods used?	No	Yes	??

(12) Statistical methods

		Correct?			Robust?			Efficient?			Relevant?		
		No	Yes	??	No	Yes	??	No	Yes	??	No	Yes	??
(a)	None												
(b)	Chi-squared / Fisher's exact test												
(c)	T-test												
(d)	Mann-Whitney / Wilcoxon												
(e)	ANOVA												
(f)	Correlation												
(g)	Regression												
(h)	Other/s (state)												
(i)	Unknown / Uncertain												

(13)	Data transformation			
	(a) Is there evidence for heterogeneity of variance?	No	Yes	??
	(b) If so were data transformed or non-parametric method used?	No	Yes	??
(14)	Number of degrees of freedom for residual?			??
(15)	Are there any obvious errors of calculation? (describe)	No	Yes	??
(16)	Graphs and tables			
	(a) Are graphs and tables clearly titled?	No	Yes	??
	(b) Are graphs and tables clearly labelled?	No	Yes	??
	(c) Are graphs appropriate (would tables be better)?	No	Yes	??
	(d) Are tables appropriate (would graphs be better)?	No	Yes	??
	(e) Are graphs or tables showing raw data for individual animals given?	No	Yes	??

(17)	Is the number of treatment groups appropriate?	No	Yes	??
(18)	Is the experimental design appropriate and does it efficiently use the resources available?	No	Yes	??
(19)	Are the conclusions drawn justified by results?	No	Yes	??
(20)	Would a different analysis make any difference to the conclusions?	No	Yes	??

(21) Other comments:
